# Leveraging artificial intelligence and machine learning to accelerate discovery of disease-modifying therapies in type 1 diabetes

**DOI:** 10.1007/s00125-024-06339-6

**Published:** 2024-12-19

**Authors:** Melanie R. Shapiro, Erin M. Tallon, Matthew E. Brown, Amanda L. Posgai, Mark A. Clements, Todd M. Brusko

**Affiliations:** 1 Department of Pathology, Immunology, and Laboratory Medicine, College of Medicine, University of Florida, Gainesville, FL USA; 2 Diabetes Institute, University of Florida, Gainesville, FL USA; 3 Division of Pediatric Endocrinology and Diabetes, Children’s Mercy Kansas City, Kansas City, MO USA; 4 Institute for Data Science and Informatics, University of Missouri-Columbia, Columbia, MO USA; 5 Department of Pediatrics, University of Missouri-Kansas City School of Medicine, Kansas City, MO USA; 6 Department of Pediatrics, College of Medicine, University of Florida, Gainesville, FL USA; 7 Department of Biochemistry and Molecular Biology, College of Medicine, University of Florida, Gainesville, FL USA

**Keywords:** Artificial intelligence, Digital twin, Drug discovery, Drug repurposing, Drug response, Immunotherapy, Machine learning, Pharmacogenetics, Precision medicine, Review, Type 1 diabetes

## Abstract

Progress in developing therapies for the maintenance of endogenous insulin secretion in, or the prevention of, type 1 diabetes has been hindered by limited animal models, the length and cost of clinical trials, difficulties in identifying individuals who will progress faster to a clinical diagnosis of type 1 diabetes, and heterogeneous clinical responses in intervention trials. Classic placebo-controlled intervention trials often include monotherapies, broad participant populations and extended follow-up periods focused on clinical endpoints. While this approach remains the ‘gold standard’ of clinical research, efforts are underway to implement new approaches harnessing the power of artificial intelligence and machine learning to accelerate drug discovery and efficacy testing. Here, we review emerging approaches for repurposing agents used to treat diseases that share pathogenic pathways with type 1 diabetes and selecting synergistic combinations of drugs to maximise therapeutic efficacy. We discuss how emerging multi-omics technologies, including analysis of antigen processing and presentation to adaptive immune cells, may lead to the discovery of novel biomarkers and subsequent translation into antigen-specific immunotherapies. We also discuss the potential for using artificial intelligence to create ‘digital twin’ models that enable rapid in silico testing of personalised agents as well as dose determination. To conclude, we discuss some limitations of artificial intelligence and machine learning, including issues pertaining to model interpretability and bias, as well as the continued need for validation studies via confirmatory intervention trials.

**Graphical Abstract:**

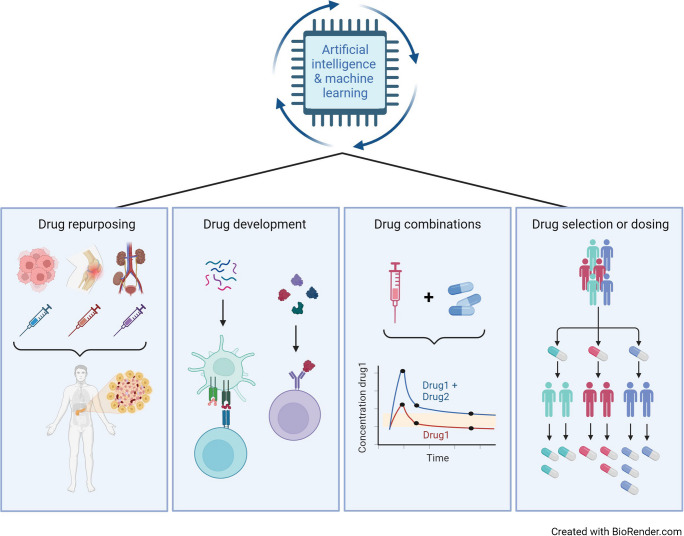

**Supplementary Information:**

The online version contains a slideset of the figures for download available at 10.1007/s00125-024-06339-6.

## Introduction

The number of disease-modifying therapies approved for slowing progression to a clinical diagnosis of type 1 diabetes remains indisputably small, despite the substantial time and resources expended toward this end. The reasons for this are multifaceted and reflect the significant challenges inherent in conducting clinical trials focused on preventing or delaying the progression of a complex chronic disease. In type 1 diabetes, factors impeding these efforts include challenges associated with identifying individuals who will rapidly progress to later-stage disease, heterogeneity in clinical response, lengthy time-to-event trial endpoints, and difficulty identifying biomarkers associated with disease progression [1–3]. More broadly, the inadequacy of existing models for evaluating candidate therapies has constrained drug discovery and development, resulting in low ‘innovation yield’ [4].

Numerous factors have been implicated in the etiopathogenesis of type 1 diabetes. Genetic risk is predominantly associated with genes that regulate immune pathways, particularly in the HLA region [5]. Enterovirus infection, dietary factors and microbiome alterations are also thought to modulate risk for type 1 diabetes [6]. However, efforts to disentangle the myriad factors involved in type 1 diabetes onset and progression have been hindered by the marked complexity and heterogeneity that characterise its epidemiology, progression and clinical presentation [7]. Of note, studies addressing factors associated with type 1 diabetes heterogeneity tend to assess individual factors (or a few of them combined), rather than assess many factors at the same time.

In November 2022, teplizumab, an anti-CD3 monoclonal antibody, was the first therapy approved by the US Food and Drug Administration for slowing progression to clinical onset of type 1 diabetes in at-risk individuals [8]. Teplizumab’s approval, which marked the first disease-modifying therapy for delaying time to diagnosis of an autoimmune disease, was the culmination of 30 years of testing in both animal models and human clinical trials, including a nearly decade-long Phase II trial [9]. The protracted, resource-intensive nature of the investigations that preceded teplizumab’s approval reflects the considerable challenges described above.

Reducing trial duration and cost hinges on the implementation of smarter trial designs and use of retrospective clinical trial data and other data generated by type 1 diabetes research networks [10–14] to identify individuals who will progress quickly to later-stage type 1 diabetes. Accomplishing these pressing needs with limited resources will be predicated on: (1) improved identification of cohorts enriched in individuals who will progress (or progress faster) to a clinical diagnosis of type 1 diabetes; (2) optimised selection and dosing of novel and repurposed therapeutic agents; and (3) improved identification of early clinical response indicators.

There is growing momentum to address these needs through team science methods that integrate advances in computational science with domain knowledge related to type 1 diabetes. Computational methods grounded in the burgeoning fields of artificial intelligence (AI) and machine learning (ML) are complementary to conventional statistical methods and can augment targeted selection of at-risk individuals for clinical trials, expedite discovery of novel therapies and dosing regimens, and improve prediction of therapeutic response [15, 16] (Table 1; see Text box, Glossary).

**Table 1:** Applications of AI/ML for accelerating translation of disease-modifying therapies to type 1 diabetes

No.	Research application	Exemplar	Exemplar pertains directly to type 1 diabetes	Exemplar description	Data source	AI/ML/informatics tool(s) or algorithm(s)	Potential future application(s) to T1D or additional applications to T1D (if exemplar pertains directly to T1D)
1	Biomarker identification	Identify proteomic biomarkers that predict progression to islet autoimmunity or clinical diagnosis of T1D by age 6 years [78]	Yes	• Used untargeted and targeted proteomics analyses to identify risk signatures associated with islet autoimmunity vs clinically diagnosed T1D	TEDDY dataset; publicly available MS data	Random forest; conditional logistic regression with LASSO	Identification of numerous types of risk biomarkers that predict progression to islet autoimmunity or to stage 1, 2 or 3 T1D
2	Multi-site cohort identification	Identify individuals previously diagnosed with various conditions [79]	No	• Used multi-site EHR data to identify individuals previously diagnosed with each of ten different conditions (e.g. heart failure, T2D)	EHR	APHRODITE	Multi-site, automated identification of cohorts of individuals diagnosed with (or at risk of developing) T1D
3	Prediction of future presence/absence of clinical disease	Identify youth who would be clinically diagnosed with T1D within 90 days [80]	Yes	• Simultaneously evaluated performance of multiple ML algorithms • Used top-performing algorithms to identify youth clinically diagnosed with T1D within 90 days	EHR	SuperLearner	Identification of individuals who will progress to islet autoimmunity or later-stage T1D
4	Evaluation of clinical trial eligibility criteria	Retrospectively evaluate the impact of clinical trial eligibility criteria on trial outcomes [81]	No	• Evaluated multi-site longitudinal data from participants in non-small-cell lung cancer trials with different eligibility criteria • Numerous eligibility criteria were found to have little-to-no impact on clinical trial outcomes	EHR data, commercial data and US Social Security Death Index data	Trial Pathfinder	Retrospective evaluation of T1D clinical trial eligibility criteria
5	Predicting adverse drug events	Calculate individual-level risk scores for experiencing various adverse events after receiving drug-modifying therapy for multiple sclerosis [82]	No	• Used temporal diagnosis, drug and measurement data from EHR • Calculated risk scores for experiencing adverse events for 12 months following exposure to disease-modifying therapy	EHR	Graph CNN; LSTM models; KG-LIME	Calculation and ranking of risk scores for experiencing adverse events following exposure to drug-modifying therapy
6	Drug target identification	Generate small molecule target predictions for known and putative cancer therapies [83]	No	• Integrated six diverse types of data • Used a Bayesian framework to identify novel small molecule targets and previously unknown targets for orphan molecules	Publicly available drug, gene expression and biological assay data	BANDIT	Identification of novel drug targets
7	Drug response prediction	Predict response to immune checkpoint inhibitor therapy [84]	No	• Analysis of clinical data from individuals with 18 types of solid tumours • Predicted response to immune checkpoint inhibitor therapy, time to cancer recurrence and life expectancy	Publicly available genomic, clinical and pathological data	LORIS	Prediction of response to drug-modifying therapy or other therapeutic interventions
8	Identification of synergistic drug combinations	Identify synergistic combinations of FDA-approved, anti-cancer therapies [85]	No	• Classified combinations of FDA-approved, anti-cancer drugs as synergistic, antagonistic or additive • Predicted effectiveness of synergistic drugs via ranked scores	Curated cancer drug dataset with associated drug combination sensitivity scores	Classification: naive Bayes, random forest, KNN, logistic regression; regression: linear, random forest and ridge regression	Identification and ranking of synergistic combinations of FDA-approved drugs that demonstrate therapeutic potential
9	Dose prediction/individualisation	Develop a highly personalised, disease- and drug-agnostic dosing methodology to optimise drug and dose selection [86]	No	• Dynamically mapped drug concentrations to phenotypic results • Provided tailored, ‘next dose’ recommendations for post-transplant immunosuppression therapy	Drug regimen, dose and trough-level data	CURATE.AI	Personalised dosing recommendations (i.e. *n*-of-1 trials) for drugs that demonstrate therapeutic potential
10	Drug repurposing	Identify candidate drugs that manipulate expression of immunomodulatory peptides involved in the pathophysiology of breast cancer [87]	No	• Evaluated deregulated immunomodulatory peptides involved in cancer regression or progression • Identified drugs that could be repurposed to alter expression of these peptides while not targeting correlated MHC genes	Publicly available scRNA-seq and ATAC-seq data	Numerous bioinformatics tools (e.g. InferCNV, Seurat and Slinky); MAST	Identify candidate drugs that manipulate expression of immunomodulatory peptides involved in the pathophysiology of T1D
11	Predicting PK/PD	Predict PD of dosing regimens containing sparse, irregularly sampled data [88]	No	• Used RNNs to model realistic, but sparse and irregularly sampled, PK/PD data • Evaluated model performance by extrapolating to unseen dosing regimens (e.g. twice daily, rather than daily)	Simulated PK/PD data	Ordinary differential equations-based RNNs, attention-based RNNs and modified RNNs	PD prediction for alternate dosing regimens that have therapeutic potential in T1D
12	Predicting progression to chronic disease	Evaluate transcriptomic signatures associated with progression to T1D [89]	Yes	• Used gene expression data from organ donors with T1D, AAb-positive individuals and control individuals to identify a T1D gene signature across islet cell types • Predicted the probability that non-diabetic AAb-positive donors would have developed T1D	Publicly available scRNA-seq data	Numerous bioinformatics tools (e.g. Seurat); Cell Ranger; support vector machine; naive Bayes; XGBoost	Computational validation of hypothesised cell–cell communication networks
13	Adaptive trial design	Evaluate interim results from a single-arm, two-stage Phase II melanoma clinical trial to facilitate design decisions for stage two of the trial [90]	No	• Evaluated interim results to determine whether the trial should continue and to select the most appropriate target response for testing drug efficacy effects • Allowed the trial sample size to be halved	Study design data found at ClinicalTrials.gov	Discrete particle swarm optimisation	Evaluation of the impact of this and other adaptive trial designs on future T1D interventional trials
14	Selection of shortened clinical endpoints	Identify transcriptomic signatures that facilitate sepsis patient stratification at a shortened endpoint [91]	No	• Retrospectively evaluated Immune Profiling Panel mRNA data • Identified high- and low-risk patient subgroups characterised by different proportions of disease worsening at a shortened endpoint	Whole-blood transcriptomic profiling data	PLS-DA	Retrospective evaluation of molecular signatures associated with shortened clinical endpoints in previous interventional trials

The selected exemplars included in this table describe AI/ML methods that can be used – or applied in new contexts – to advance the discovery of disease-modifying therapies in type 1 diabetes. Many other examples of these and other AI/ML applications, tools and algorithms have been published. Some exemplars listed in this table pertain directly to type 1 diabetes; others are potentially relevant but do not directly pertain to type 1 diabetes. The ‘Data source’ column notes the type of data used to carry out each study; however, AI/ML methods are generally agnostic to data type and thus can be carried out with other types of data. Note: Although AI/ML methods use sophisticated pattern recognition techniques to map data inputs to observed outputs, these methods should not be interpreted as causal analyses

APHRODITE, Automated PHenotype Routine for Observational Definition, Identification, Training and Evaluation; ATAC-seq, assay for transposase-accessible chromatin using sequencing; BANDIT, Bayesian ANalysis to determine Drug Interaction Targets; CNN, convolutional neural network; EHR, electronic health records; FDA, US Food and Drug Administration; KG-LIME, knowledge graph local interpretable model agnostic explanation; KNN, k-nearest neighbours; LASSO, least absolute shrinkage and selection operator; LORIS, Logistic Regression-Based Immunotherapy-Response Score; LSTM, long short-term memory; MAST, Model-based Analysis of Single-cell Transcriptomics; PD, pharmacodynamics; PK, pharmacokinetics; PLS-DA, partial least squares discriminant analysis; RNN, recurrent neural network; scRNA-seq, single-cell transcriptomics; T1D, type 1 diabetes; T2D, type 2 diabetes; TEDDY, The Environmental Determinants of Diabetes in the Young; XGBoost, eXtreme Gradient Boosting


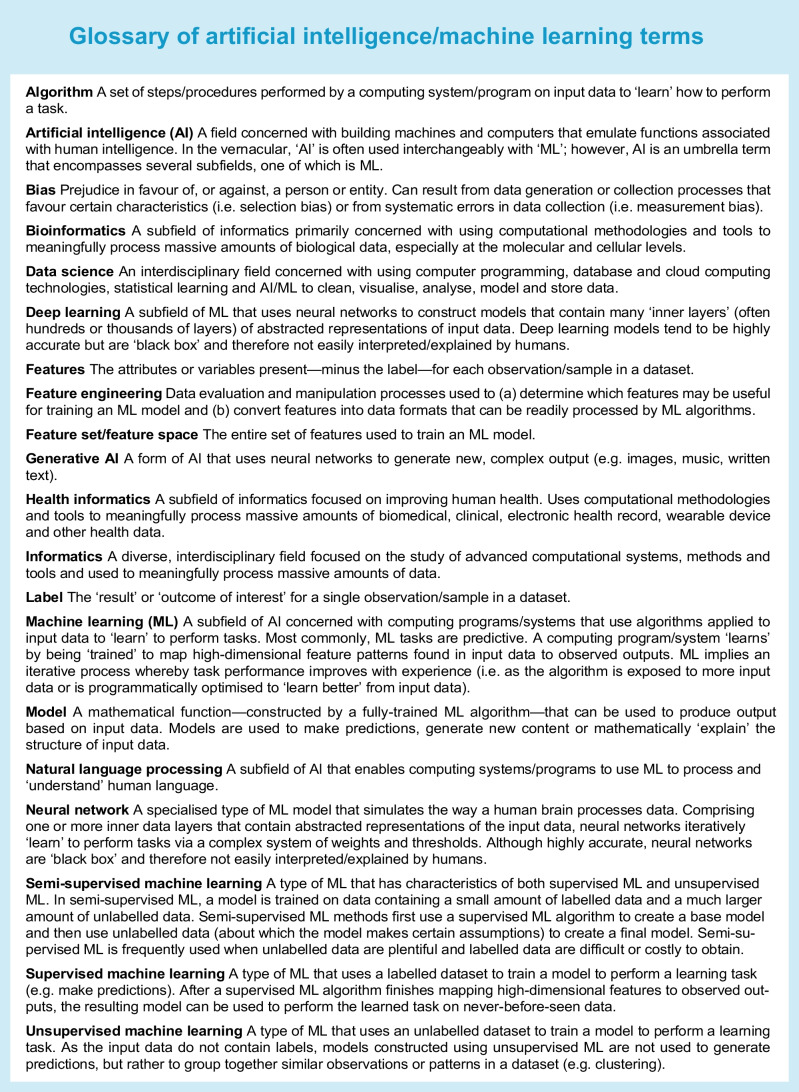


Here, we review ground-truth concepts for disease progression and the emerging role of biologically informed, AI/ML-enabled analytics in advancing immunogenetic discovery and accelerating the development and translation of new disease-modifying therapies in type 1 diabetes.

### Current status of type 1 diabetes knowledge discovery: conventional statistical methods

Current knowledge discovery efforts generally employ conventional statistical methods to make inferences from data through the estimation of unknown parameters or probability distributions [17]. These methods are well established, can be used in a wide variety of research contexts and have facilitated countless discoveries in diabetes research. Proportional hazards regression models have been used, for example, to evaluate the ability of novel composite measures such as Index60 to aid identification of individuals who will progress more quickly to clinical type 1 diabetes [18]. However, such indices are based solely on type 1 diabetes domain knowledge and account for only a very limited number of input variables [18].

Conventional statistical methods should, broadly speaking, be used only with datasets containing a limited number of observations, variables and interaction terms [17]. These methods also assume a priori knowledge about the shape and distribution of the data and about variables thought to be related to the outcome of interest [17, 19]. Overall, the utility of these methods is constrained by their insufficient ability to handle diverse data types, strong a priori assumptions, scalability considerations and limited applicability to the discovery of personalised treatment options and dosing regimens [19].

### Emerging capabilities in type 1 diabetes knowledge discovery: AI/ML

**Fig. 1:**
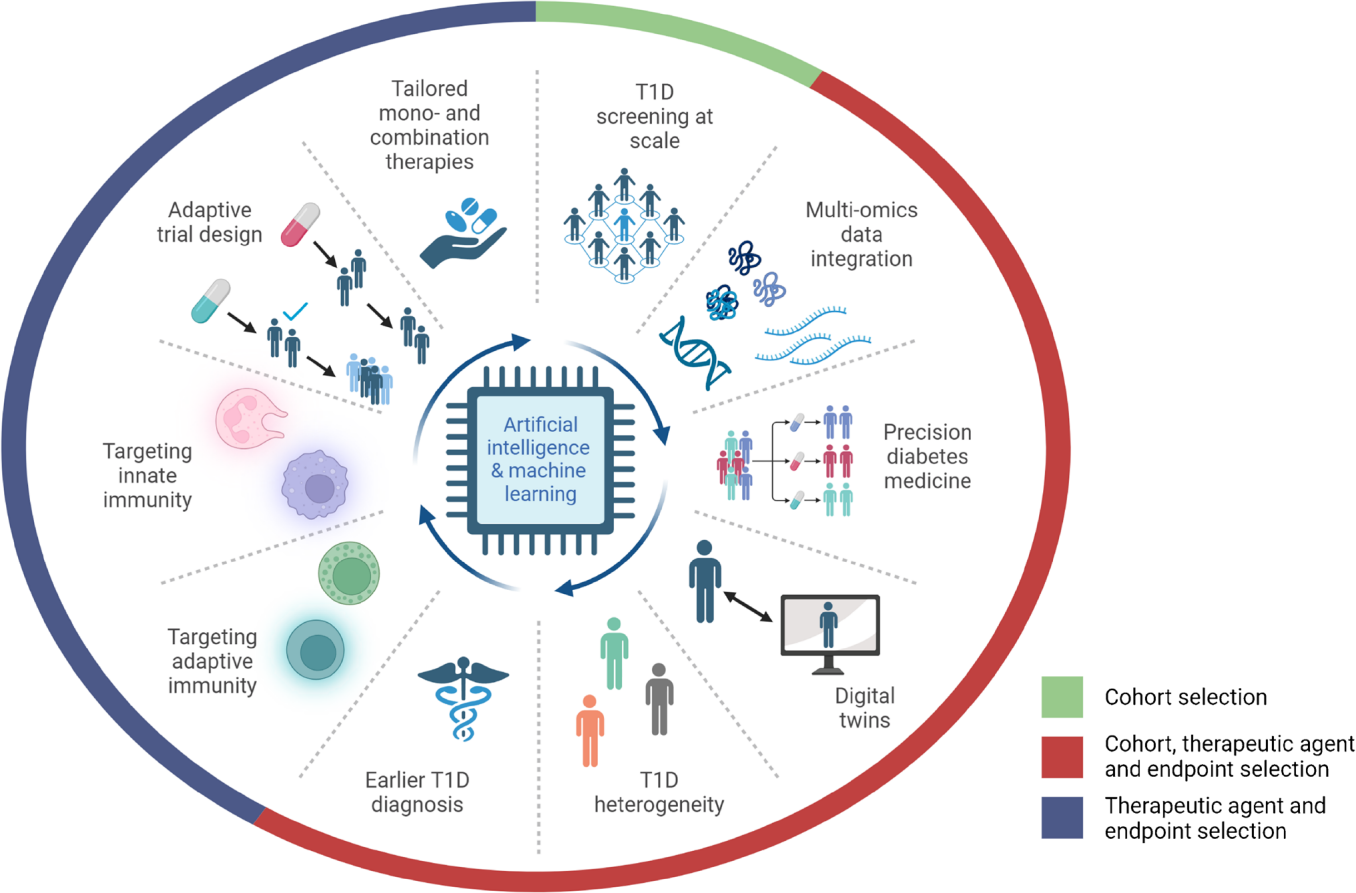
Leveraging AI/ML for smarter trial design. The increasing availability of diverse, high-dimensional datasets and advanced computing technologies is providing opportunities to use AI/ML to model complex phenotypes and biological phenomena at scale. The AI/ML applications depicted here—when informed by domain expertise in type 1 diabetes—are poised to generate substantial gains in the development and translation of disease-modifying therapies for type 1 diabetes. To this end, AI/ML can be effectively leveraged to optimise cohort, therapeutic agent and clinical endpoint selection. T1D, type 1 diabetes. Created with BioRender.com. This figure is available as part of a downloadable slideset

Recent advances in engineering and computational science have profoundly impacted the type and scale of digitised health data now available for research, as well as the development of AI/ML methods for meaningfully processing large, complex datasets [16, 20]. These data include high-dimensional omics (e.g. genomic and proteomic) data, as well as data collected via electronic health records, multiple clinical trials targeting similar outcomes (e.g. progression to later-stage type 1 diabetes), surveys, fitness wearables, mobile apps, glucose monitors and insulin delivery devices [20]. The simultaneous availability of cloud and high-performance computing technologies optimised to work with high-dimensional data presents diabetes researchers with new opportunities to collaborate with computational scientists who possess the technical skillsets needed to work with ‘big data’ (Fig. 1).

Because AI/ML methods can detect higher-order, non-linear relationships in high-dimensional data, these methods can be used to identify essential data patterns and then collate them via complex models for forecasting outcomes [16, 21]. Importantly, excitement generated by AI/ML has, at times, resulted in the perception that these methods have a near-magical ability to ‘learn’ and generate meaningful output [22, 23]. However, data type and quality matter, and the maxim ‘garbage in, garbage out’ indiscriminately applies to diabetes-related applications of AI/ML [22]. Transdisciplinary domain expertise and a commitment to methodological rigor necessarily and meaningfully impact the development, interpretation and validation of AI/ML models.

Today, AI/ML technologies are already being used to develop digital twin models (i.e. to virtually test interventions under various conditions), discover novel drug targets and identify biomarker signatures of disease risk and progression [21]. By facilitating virtual testing of multiple therapeutic agents in silico, these technologies are poised to impact the evidence base informing whether the field should invest in trials of specific agents, as well as raise the possibility of using only virtual – or even no – placebo groups [15, 24].

## Drug discovery, development and selection

### Repurposing active agents from other diseases

**Fig. 2:**
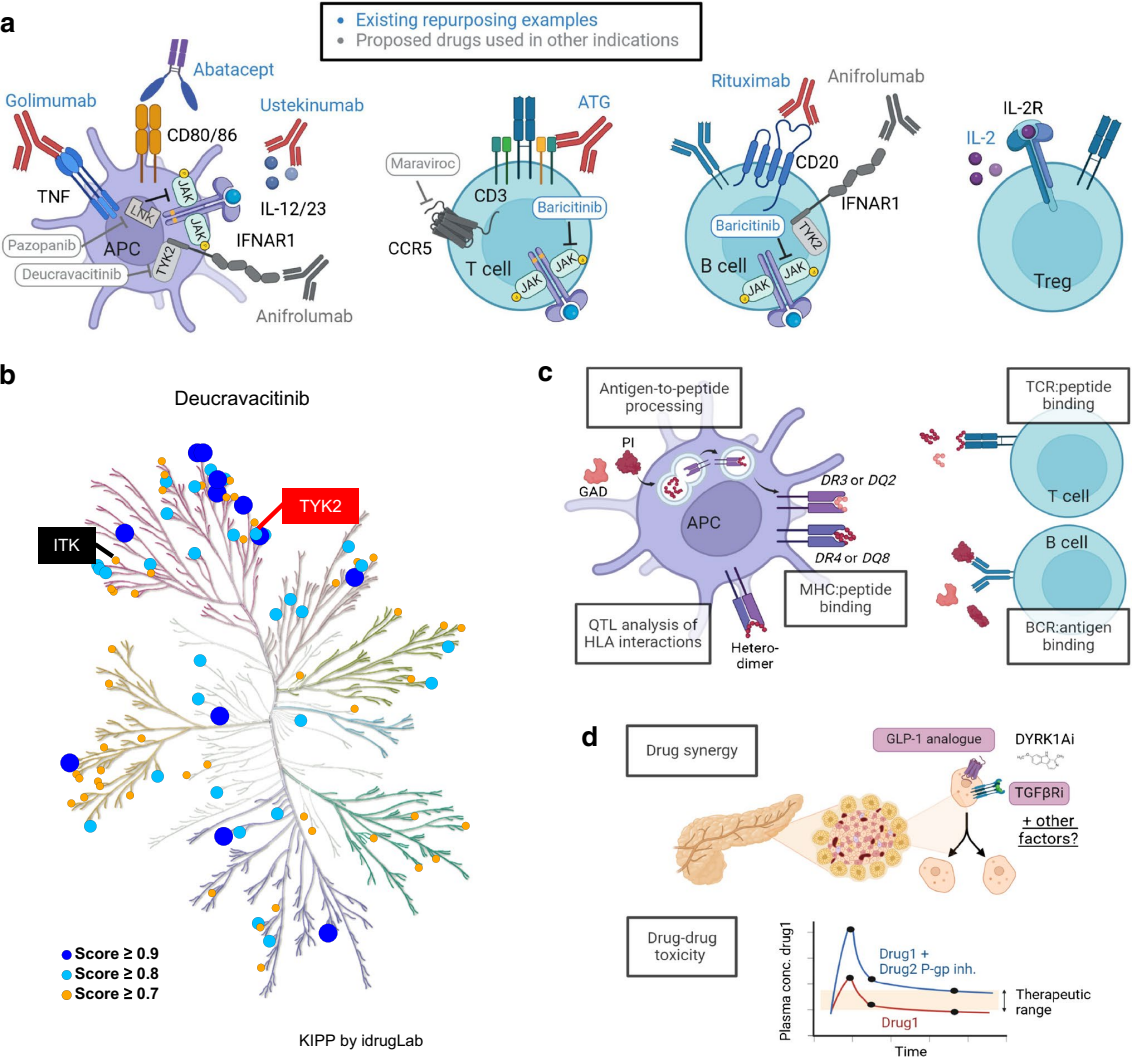
Application of AI/ML to drug repurposing, development and combination therapies. (**a**) Drugs that have been previously repurposed from other diseases to type 1 diabetes are shown in blue, including the antigen-presenting cell (APC) regulators golimumab (anti-TNF), abatacept (CTLA-4-Ig) and ustekinumab (anti-IL-12p40); the T cell deplete, ATG (anti-thymocyte globulin); the B cell deplete, rituximab (anti-CD20); the cytokine signalling inhibitor, baricitinib (JAK1/2 blockade); and the Treg enhancer, low-dose IL-2. Drugs that we propose for repurposing in type 1 diabetes are shown in grey, including the cytokine/chemokine signalling inhibitors anifrolumab (anti-IFNAR1), pazopanib (off-target LNK/SH2B3 blockade), deucravacitinib (TYK2 blockade) and maraviroc (CCR5 blockade). (**b**) Example of ML-powered kinase profiling prediction showing predicted drug–target binding scores. Kinase tree diagram showing that deucravacitinib inhibits TYK2. The kinome-wide inhibitory predict platform (KIPP) identified an off-target effect of deucravacitinib for a GWAS hit related to type 1 diabetes, ITK (IL-2 inducible T cell kinase). Created with KIPP by idrugLab [33]. (**c**) Opportunities for use of AI/ML algorithms in the design of antigen-specific immunotherapies for type 1 diabetes include models for antigen-to-peptide processing, MHC:peptide binding, QTL analysis of HLA associations, T cell receptor (TCR):peptide binding, and B cell receptor (BCR):antigen binding. PI, proinsulin. (**d**) Opportunities for AI/ML use in selecting combination therapies include models of drug synergy and drug–drug toxicity. We propose using synergy models to identify factors that can augment in vivo beta cell proliferation beyond that seen with glucagon-like peptide-1 (GLP-1) analogues, dual-specificity tyrosine phosphorylation-regulated kinase 1A inhibitors (DYRK1Ai) and TGF-β receptor inhibitors (TGFβRi). Drug toxicity can occur when drug efflux transporters are inhibited, thereby increasing intracellular concentrations of a second drug. CCR5, C-C motif chemokine receptor 5; IFNAR1, IFN alpha and beta receptor subunit 1; JAK, janus kinase; LNK (also known as SH2B3), SH2B adaptor protein 3; P-gp inh., P-glycoprotein inhibitor; TYK2, tyrosine kinase 2. Created with BioRender.com. This figure is available as part of a downloadable slideset

To accelerate time to trial, most immunotherapies trialled in the type 1 diabetes intervention space have been repurposed from transplantation, cancer and other autoimmune diseases. These include agents that: (1) deplete or inhibit activation of T cells or B cells; (2) block inflammatory cytokines or downstream signalling; or (3) enhance regulatory T cell (Treg) numbers and/or suppressive capacity (Table 2, Fig. 2a). The rationale for some therapies is informed by specific, known genetic risk loci for type 1 diabetes (e.g. *CTLA4* and *IL2RA*), suggesting the potential for differential responsiveness among participants. Abatacept, for example, is administered for cytotoxic T-lymphocyte-associated protein 4 (CTLA-4) rescue [25, 26], and low-dose IL-2 is used to overcome IL-2 and/or IL-2 receptor subunit alpha (IL-2RA) deficiencies [27, 28]). Other therapies have been proposed to address more generalised mechanisms of type 1 diabetes pathogenesis due to blockade of multiple targets (e.g. polyclonal anti-thymocyte globulin [ATG] [29], janus kinase (JAK) inhibition by baricitinib affecting broad cytokine signalling [30]). Despite the success of many of these agents in attaining primary endpoints, all tested interventions have only transiently preserved endogenous C-peptide compared with placebo, suggesting a need for intervention at earlier disease stages, continuous therapy or re-treatment, establishment of combination therapies and/or identification of single agents with longer-lasting effects.

**Table 2:** Repurposed immunotherapeutic drugs for treating type 1 diabetes

Mechanism of action	Drug	Target	Approved indications	T1D GWAS genes	T1D GWAS lead variant	T1D trial phase	T1D clinical endpoint	Endpoint significant	Reference
Inhibit T cell activation	Abatacept (CTLA-4-Ig)	CD80/CD86	RA, JIA, PsA, aGVHD	*CTLA4*	rs3087243	Phase II	2 h MMTT at 2 years	Yes, *p*=0.0029	[26]
						Phase II	Time to AGT in stage 1 at-risk FDRs	No, *p*=0.11	[25]
	Alefacept (LFA-3-Ig)	CD2	PsO	N/A	N/A	Phase II	2 h MMTT at 1 year	No, *p*=0.065	[92]
							2 h MMTT at 2 years	Yes, *p*=0.015	[93]
Deplete T or B cells	Low-dose ATG	Polyclonal	Transplant	*CD3G*	rs3753059	Phase II	2 h MMTT at 1 year	Yes, *p*=0.00005	[29]
				*IL2RA*	rs12722496				
				*CTLA4*	rs3087243				
				HLA class I	Several tag SNPs				
				HLA class II	Several tag SNPs				
	Rituximab	CD20	Cancer, RA, AAV, PV	N/A	N/A	Phase II	2 h MMTT at 1 year	Yes, *p*=0.03	[57]
Block inflammatory cytokines or downstream signalling	Baricitinib	JAK1/2	RA, AA	*TYK2*	rs34536643	Phase II	2 h MMTT at 48 weeks	Yes, *p*=0.001	[30]
	Golimumab	TNF	RA, PsA, AS	*TNFAIP3*	rs6918329	Phase II	4 h MMTT at 1 year	Yes, *p*<0.001	[94]
	Ustekinumab	IL-12p40	PsA, PsO, IBD	N/A	N/A	Phase I/II	Safety	Yes	[95]
Enhance Treg numbers and/or suppressive capacity	Low-dose IL-2	IL-2R	Cancer	*IL2*	rs3136534	Phase I/II	Dose-dependent changes in Treg:CD4^+^ and Treg CD25 MFI	Yes, *p*<0.0001	[27]
				*IL2RA*	rs12722496	Phase I/II	Dose-dependent change in Treg	Yes, *p*=0.0002	[28]

Mechanism of action, target, approved indications, relations with type 1 diabetes-associated genes and variants, trial phase and results of the primary clinical endpoints are presented for each drug. Lead variants for type 1 diabetes-associated genes were obtained from the Type 1 Diabetes Knowledge Portal (RRID:SCR_020936) in June 2024. Drugs without known impacts on type 1 diabetes-associated genes are shown as N/A

AA, alopecia areata; AAV, anti-neutrophilic cytoplasmic antibody (ANCA)-associated vasculitis; AGT, abnormal glucose tolerance; aGVHD, acute graft vs host disease; AS, ankylosing spondylitis; FDR, first-degree relative; GWAS, genome-wide association study; IBD, inflammatory bowel disease; JIA, juvenile idiopathic arthritis; LFA-3, lymphocyte function-associated antigen 3; MFI, mean fluorescence intensity; MMTT, mixed meal tolerance test; PsA, psoriatic arthritis; PsO, psoriasis; PV, pemphigus vulgaris; RA, rheumatoid arthritis

Future immunotherapy efforts in type 1 diabetes should implement algorithms designed to identify existing drugs that affect targets of interest (Table 1, nos 6 and 10). AI/ML-based methods for drug repurposing incorporate genomic and transcriptomic data, chemical structures and existing drug–target interaction knowledge to make novel drug–target, drug–cell/tissue and/or drug–disease predictions [31]. To demonstrate the utility of existing drug repurposing databases, we surveyed the Drug Repurposing Hub [32] for genes associated with type 1 diabetes risk variants to identify drugs with demonstrated on- or off-target effects in other indications (Table 3, Fig. 2a). To demonstrate the use of ML in predicting off-target effects of existing drugs, we generated a kinase profiling prediction model [33] that highlights experimentally validated on-target and predicted off-target type 1 diabetes genome-wide association (GWAS)-identified hits for a small molecule kinase inhibitor (Fig. 2b). At the time of this review, we have confirmed that none of these agents is undergoing a registered clinical trial for type 1 diabetes [34] and that minimal preclinical data exist for most of these agents in the context of type 1 diabetes.

**Table 3:** Proposed immunotherapeutic drugs to repurpose for treating type 1 diabetes

Mechanism of action	Drug	Target	Approved indications	T1D GWAS genes	T1D GWAS lead variant	Reference
Block inflammatory cytokines, chemokines or downstream signalling	Anifrolumab	IFNAR1	SLE	*TYK2*	rs34536643	[96]
				*IFIH1*	rs2111485	
	Deucravacitinib	TYK2	PsO	*TYK2*	rs34536643	[97]
	Maraviroc	CCR5	HIV	*CCR5*	rs57319220	[98]
Inhibit tyrosine kinases	Pazopanib	VEGFR, PDGFR, KIT	Cancer	*SH2B3*	rs7137828	[99]
	Vandetanib	VEGFR, EGFR, RET	Cancer	*ERBB3*	rs1131017	[100]

For each drug, the mechanism of action, target, approved indications, and relations with type 1 diabetes-associated genes and variants are presented. Lead variants for type 1 diabetes-associated genes were obtained from the Type 1 Diabetes Knowledge Portal (RRID:SCR_020936) in June 2024

CCR5, C-C motif chemokine receptor 5; EGFR, EGF receptor; GWAS, genome-wide association study; IFNAR1, IFN alpha and beta receptor subunit 1; KIT, KIT proto-oncogene, receptor tyrosine kinase; PDGFR, platelet derived growth factor receptor; PsO, psoriasis; RET, ret proto-oncogene; SLE, systemic lupus erythematosus; TYK2, tyrosine kinase 2; VEGFR, vascular endothelial growth factor receptor

### Antigen-specific immunotherapies

Early evidence of antigen-specific immunotherapies promoting C-peptide maintenance in subgroups of clinical trial participants with or at risk for developing type 1 diabetes supports future efforts to use AI/ML to identify responder signatures and novel targets (Table 1, nos 6, 7 and 9). In support of disease endotype concepts, response to GAD65-alum treatment has been linked to the presence of *HLA*-*DR3*-*DQ2* in GAD65 autoantibody (AAb)-positive recent-onset participants [35, 36], while oral insulin showed benefit in a subgroup of participants with high IAA titres [37]. Using this knowledge, proinsulin therapies have been designed using an immunodominant *HLA*-*DR4*-restricted peptide [38] with the consideration that those carrying this HLA risk haplotype may respond best because of their tendency for initial IAA seroconversion [39]. Recent advances in incorporating AI/ML-based decision-making into quantitative trait locus (QTL) analysis [40] may further elucidate the influence of additional HLA genetics on response to antigen therapies by permitting modelling of complex non-additive effects (Fig. 2c).

We envision that knowledge of HLA genetics associated with response to antigen therapy will inform algorithms used to predict peptides most likely to result from antigen processing and to be loaded onto particular HLA specificities [41]. In this regard, unbiased immunopeptidome studies using methods capable of detecting post-translational modifications [42] will be important to incorporate into such algorithms to identify actionable target neoepitopes for participant subgroups. As proof of concept, novel peptides derived from beta cell secretory granule proteins were recently identified via HLA class I peptidomics, using predicted peptide–HLA binding to prioritise peptides for experimental validation [43]. From the antigen recognition side, while additional B cell receptor and T cell receptor sequences enriched in type 1 diabetes continue to be discovered using state-of-the-art AI/ML-based approaches (i.e. ImmuneML [44]) and curated in public databases (i.e. iReceptor [45]), we anticipate that these data may also facilitate prediction of novel antigen and peptide specificities [46] for tolerance induction (Fig. 2c).

### Combination therapies

While single agents have shown success in transiently maintaining endogenous C-peptide levels in recent-onset type 1 diabetes [47], extending the duration of therapeutic efficacy may require consideration of combination therapies (Table 1, no. 8). A clinical trial of α-IL-21 plus liraglutide [48], which each address distinct pathogenic mechanisms, showed success by simultaneously inhibiting inflammation and directly affecting beta cell function. Accelerating the discovery of novel synergistic combination therapies while avoiding toxicity due to drug interactions is likely to require moving beyond such hypothesis-driven ideas toward in silico prediction methods (Table 1, no. 7).

AI/ML approaches are beginning to be incorporated into physiologically based pharmacokinetic models capable of estimating drug toxicity via effects on drug absorption, distribution, metabolism and/or excretion [49]. One can appreciate how these new capabilities might have been able to predict the past failure of rapamycin plus IL-2 for type 1 diabetes prevention [50], had they been available at the time. Given previous knowledge that low-dose IL-2 inhibits the drug efflux pump, P-glycoprotein [51], the combination may have increased in vivo rapamycin concentrations, thereby promoting beta cell toxicity (Fig. 2d). Future studies evaluating potential combination therapies should use both in vitro and in silico methods to pre-emptively advise about adverse drug–drug interactions and guide informed and perhaps altered agent selection or dosing decisions.

Prediction of drug–drug interactions also includes the potential for drug synergism discovery, which has shown progress in cancer applications [52]. These tools rely on integration of known effects of drug combinations at various doses on the viability of cancer cell lines, with prediction of novel drug–drug synergism according to drug–target interactions in shared pathways [52]. While inhibitors of the cell cycle regulator dual-specificity tyrosine phosphorylation-regulated kinase 1A (DYRK1A) have been successfully combined with TGF-β inhibitors or glucagon-like peptide-1 (GLP1) analogues to promote human beta cell transdifferentiation from alpha cells, further enhancing in vivo beta cell numbers with additional drugs may be necessary for clinical effect in established type 1 diabetes [53] (Fig. 2d). Likewise, combinations of immunosuppressive drugs have been shown to effectively and safely expand Tregs in solid organ transplantation [54], with potential translation to type 1 diabetes. Thus, in silico prediction of effective drug combinations and doses based on in vitro drug screens [55] may inform strategies for beta cell regeneration and amelioration of autoimmunity in type 1 diabetes.

## Responder identification and implications for precision medicine

In addition to identifying, repurposing or combining drugs for type 1 diabetes at the population level, novel computational methods may inform precision medicine efforts to select optimal drugs and/or dosing based on patient characteristics (Table 1, nos 1, 3, 4, 7, 9, 13 and 14). Conventional statistical methods have been successfully used in at-risk and recent-onset type 1 diabetes cases to identify immune signatures of immunotherapy responders, as summarised in a previous review [56]. However, an important caveat is that the majority of these signatures appear after treatment and therefore they are not inherently predictive of response [56]. This highlights a need for: (1) identification of characteristics that can distinguish response prior to treatment or (2) a means to model and predict individuals’ responsiveness in silico or in vitro.

### Demographics

Rituximab and ATG tend to have a greater impact on C-peptide maintenance in younger [57] and older participants [58], respectively. While abatacept was previously shown to have a negative impact on C-peptide maintenance in individuals with recent-onset type 1 diabetes who were from racial and ethnic minority groups [26], social determinants of health often preclude such individuals from participation in clinical trials, lending uncertainty to the generalisability of this finding to other immunotherapies in type 1 diabetes. Trials with extended age ranges and more diverse cohorts are needed to validate these findings before incorporating them into predictive algorithms. Additionally, when considering generalisability and response prediction for antigen-specific immunotherapies, ancestry-specific risk HLAs for type 1 diabetes [5] must be considered.

### Genetics

**Fig. 3:**
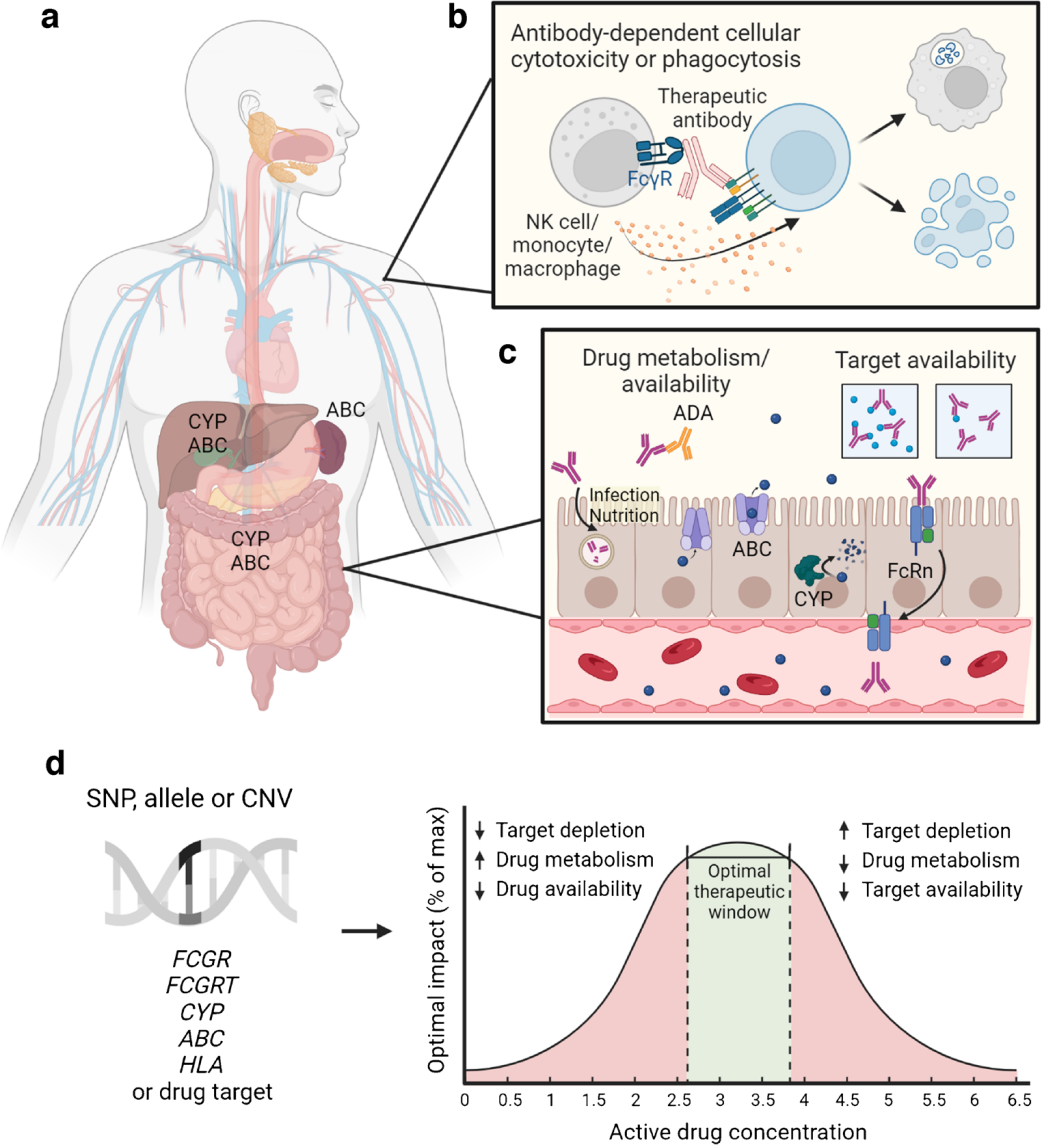
Responder identification and potential for AI-enabled precision medicine. (**a**) Algorithms for predicting drug metabolism and/or availability incorporate information about genetic variants affecting drug metabolism via cytochrome P450 (CYP) enzymes and efflux via ATP-binding cassette (ABC) transporters. While not yet incorporated into drug response algorithms, we propose that (**b**) SNPs and copy number variants (CNVs) affecting FcγR may aid in predicting response to therapeutic antibodies. (**c**) HLA-associated development of anti-drug antibodies (ADA), neonatal Fc receptor for IgG (FcRn)/*FCGRT* variants, and environmental factors including infection and malnutrition regulate drug catabolism, which may also affect drug availability. (**d**) Together, these variants may inform drug dosing or selection by influencing target depletion, drug metabolism or drug availability. NK, natural killer cell. Created with BioRender.com. This figure is available as part of a downloadable slideset

While prediction of drug responsiveness in most other applications has focused on genetic variants, there have been few reports in type 1 diabetes beyond an association between the *HLA*-*DR4*^+^*HLA*-*DR3*^−^ genotype and increased response to teplizumab in at-risk individuals (stage 2 type 1 diabetes) [59]. Efforts thus far have been limited to analysis of known type 1 diabetes risk genes with minimal, if any, application of pharmacogenetics knowledge. Post hoc analysis of small molecule drugs in type 1 diabetes should use physiologically based pharmacokinetic models that have been developed to incorporate information about alleles of cytochrome P450 (CYP) family genes, which play a role in drug metabolism via oxidation, or ATP-binding cassette (ABC) family genes [49, 60], which are responsible for drug efflux, both of which could potentially inform individualised dosing. Effective dosing of therapeutic antibodies, which bypass first-pass metabolism [61], may be influenced by variants modulating the affinity of Fcγ receptors (FcγR) for the Fc portion of IgG, thereby influencing mechanisms of action including antibody-dependent cellular cytotoxicity and antibody-dependent cellular phagocytosis, as well as by neonatal Fc receptor for IgG variants affecting antibody trafficking [62]. The development of anti-drug antibodies, which may be influenced by HLA genetics, presents an additional factor that might confound drug bioavailability [63]. Beyond individual genetics, environmental factors such as infection or malnutrition can impart systemic inflammation that speeds up the breakdown of monoclonal antibodies [64] (Fig. 3). As AI/ML models begin to integrate these parameters for the prediction of therapeutic antibody response and pharmacokinetics, this may inform precision dosing strategies that both optimise efficacy and attenuate adverse effects.

Methods capable of predicting resistance to therapies may also aid in individualised active agent selection [65]. Studies from other diseases have identified genetic variants that affect drug-specific mechanisms for agents that have been repurposed with some success in the type 1 diabetes space. For example, an *IL12B* expression QTL (eQTL) leading to reduced IL-12 expression has been linked to poor response to ustekinumab in psoriasis [66]. Likewise, a *TNF* SNP causing decreased TNF levels was significantly associated with decreased response to golimumab in Behçet’s syndrome [67]. Beyond these direct examples, variants affecting the function or expression of proteins involved in downstream signalling pathways or more complex crosstalk between pathways may also affect drug efficacy in a less easily predictable manner. Here, the use of ‘digital twin’ technology incorporating precision genetics could permit in silico testing of several immunotherapies to allow individualised selection of the most potentially efficacious agent, dosing, etc. [21]. While immune-based ‘digital twin’ models are currently in their infancy, the concept is important considering the challenges of translating in vitro responsiveness at the cell or tissue level into personalised predictions of in vivo drug efficacy. We suggest using a standard metric of clinical response based on historical trial data (e.g. the quantitative response – QR value [68]) as a predicted outcome of treatment with different agents and/or dosing based on individual clinical, immunological or genetic characteristics, permitting eventual validation in trials that recruit predicted responder cohorts.

## AI/ML limitations and considerations

In this section we highlight the challenges and considerations underscoring the clinical relevance, ethical development and equitable implementation of AI/ML in type 1 diabetes therapeutic discovery.

### Novelty vs relevance

Numerous factors impact the actionability, generalisability, replicability and interpretation of AI/ML results. For example, data cleaning and transformation tasks must account for processes used during data collection (e.g. study inclusion/exclusion criteria and data collection time windows) [22]. When data are transformed for supervised and semi-supervised AI/ML, researchers and model developers must work together to ensure that models are constructed using accurately labelled, ground-truth datasets that reflect the nature of the reality being modelled [16]. Without active engagement with diabetes domain experts, developers can easily misunderstand models’ clinical actionability by, for example, evaluating models using only metrics that emphasise model performance, rather than considering a model’s ability to augment clinical decision-making [69].

To meaningfully engage in and critically appraise research involving AI/ML methods, type 1 diabetes domain experts and clinicians must prioritise increasing their AI/ML literacy, knowledge of factors that adversely impact AI/ML model performance and validation (e.g. overfitting), and understanding of data management workflows and the data life cycle [69, 70]. Interdisciplinary research is not new to type 1 diabetes research, which has historically bridged paediatric and adult endocrinology, immunology, histopathology, genetics, microbiology, cellular and molecular biology, biomedical engineering and more. Team science initiatives that bridge knowledge gaps between clinicians, researchers and data scientists will be critical to improving the relevance of AI/ML research findings in our field.

### Explainability

Many of the most powerful AI/ML methods, such as deep learning, are computational ‘black boxes’ that cannot be readily explained by humans [69, 71]. This lack of explainability will constrain efforts to determine whether model predictions align with type 1 diabetes domain expertise [71].

As a result, significant research efforts are now focused on improving the explainability of AI/ML models [72]. Referred to as explainable AI (XAI), these methods—currently used primarily in the context of supervised and semi-supervised AI/ML—emphasise the use of human-interpretable ML approaches (e.g. decision tree-based models) and post hoc approaches for explaining opaque predictions (e.g. methods for calculating the importance of complex features used to generate predictions) [73]. Human-in-the-loop and knowledge domain approaches represent additional promising approaches for integrating human expertise into model development and interpretation processes [72]. Given the non-trivial implications of AI/ML-enabled analytics in type 1 diabetes research and clinical care, incorporating XAI approaches will be key to earning users’ trust, aiding error identification efforts (e.g. rejecting biologically implausible findings) and enhancing informed decision-making [72, 73].

### Bias

Although AI/ML models constructed using massive amounts of data should theoretically approximate objective truth, massive datasets often contain hidden biases [69, 74]. AI/ML models are not capable of intrinsically ‘knowing’ these biases and cannot reason about their causes in the same way that human experts can [22, 74]. Models created from such data are prone to generating predictions that systematically underperform for individuals, observations or samples impacted by these biases—often unbeknown to human experts [69, 75]. Investigators must therefore commit to: (1) including ancestrally diverse, representative samples in training data; (2) critically evaluating model results in light of biases known to impact AI/ML models; (3) externally validating models using methods appropriate to the size and type of training data used; and (4) following reporting guidelines that enhance model reproducibility and transparency [69, 75].

### FAIR principles and data standards

Stewarding scientific data and other digital research objects (e.g. algorithms, computational tools and workflows) necessitates ensuring that these resources are Findable, Accessible, Interoperable and Reusable (FAIR) [76]. The FAIR principles ensure that these digital research objects are maximally transparent and reproducible. These principles emphasise the importance of metadata, which refers to detailed information describing the ‘how, what and why’ of digital research objects. High-quality metadata are key to lowering barriers to data reuse and ensuring transparency and reproducibility when data contain highly sensitive or individually identifiable information [23]. The FAIR principles would, for example, support publication of metadata outlining key demographic and medical information, thus aiding the interpretation of individual (or, in sensitive contexts, summary-level) type 1 diabetes genotyping data from the Database of Genotypes and Phenotypes (dbGaP).

A wealth of geographically diverse type 1 diabetes data has been collected in numerous longitudinal studies. However, between-study differences in inclusion/exclusion criteria, data collection and data documentation processes have made it difficult to reconcile differences across disparate datasets. Numerous data standards (e.g. adaptive immune receptor repertoire standards) have been designed to ‘adjust’ for structural variations in the way that data are collected, stored and exchanged [77]. These standards facilitate data interoperability and transformation of data into features that can be used for algorithm training, as well as external model validation [22, 76]. We encourage type 1 diabetes researchers to incorporate these data standards into their work and to avail themselves of resources now available for data FAIRification [76].

## Conclusion

We are on the cusp of an emerging state of knowledge discovery in type 1 diabetes where biologically informed AI/ML-enabled analytics can facilitate drug discovery/reassignment, expedite cohort selection, foster development of smarter trial designs and optimise therapeutic response prediction. In this emerging paradigm, AI/ML—used alongside conventional statistical methods—may enable the identification of mechanistic biomarkers and surrogate endpoints that drive earlier interrogation of efficacy, shorten trial timelines and potentially reduce or even eliminate the need for treatment control participants. We envision a future research landscape where AI/ML facilitates the development of a connected health data ecosystem that promotes participatory, data-driven, person-centred health.

## Supplementary Information

Below is the link to the electronic supplementary material.

Slideset of figures (PPTX 0.99 MB)

## Abbreviations

AAb: Autoantibody
ABC: ATP-binding cassette
AI: Artificial intelligence
ATG: Anti-thymocyte globulin
CTLA-4: Cytotoxic T-lymphocyte-associated protein 4
FAIR: Findable, Accessible, Interoperable and Reusable
FcγR: Fcγ receptor
GWAS: Genome-wide association study
JAK: Janus kinase
ML: Machine learning
QTL: Quantitative trait locus
Treg: Regulatory T cell
XAI: Explainable artificial intelligence
