# Supplementary material for: Leveraging artificial intelligence and machine learning to accelerate discovery of disease-modifying therapies in type 1 diabetes
Source: Diabetologia. 2024 Dec 19;68(3):477–94. doi: 10.1007/s00125-024-06339-6 (PMC11832708; doi:10.1007/s00125-024-06339-6)
Supplement: Supplementary file 1 — Slideset of figures (PPTX 0.99 MB) [file 125_2024_6339_MOESM1_ESM.pptx]

## Slide 1
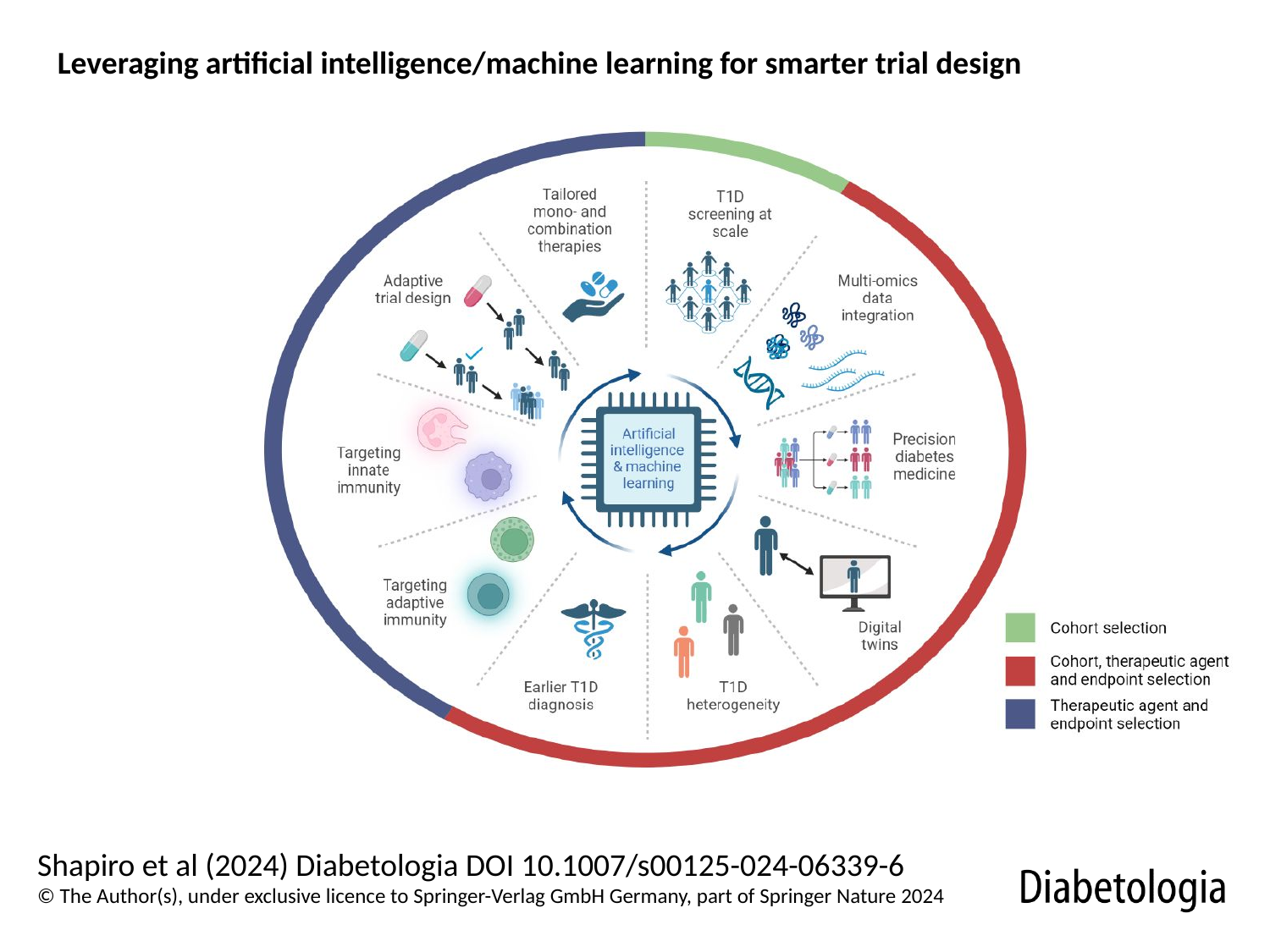

Leveraging artificial intelligence/machine learning for smarter trial design
Shapiro et al (2024) Diabetologia DOI 10.1007/s00125-024-06339-6
© The Author(s), under exclusive licence to Springer-Verlag GmbH Germany, part of Springer Nature 2024

## Slide 2
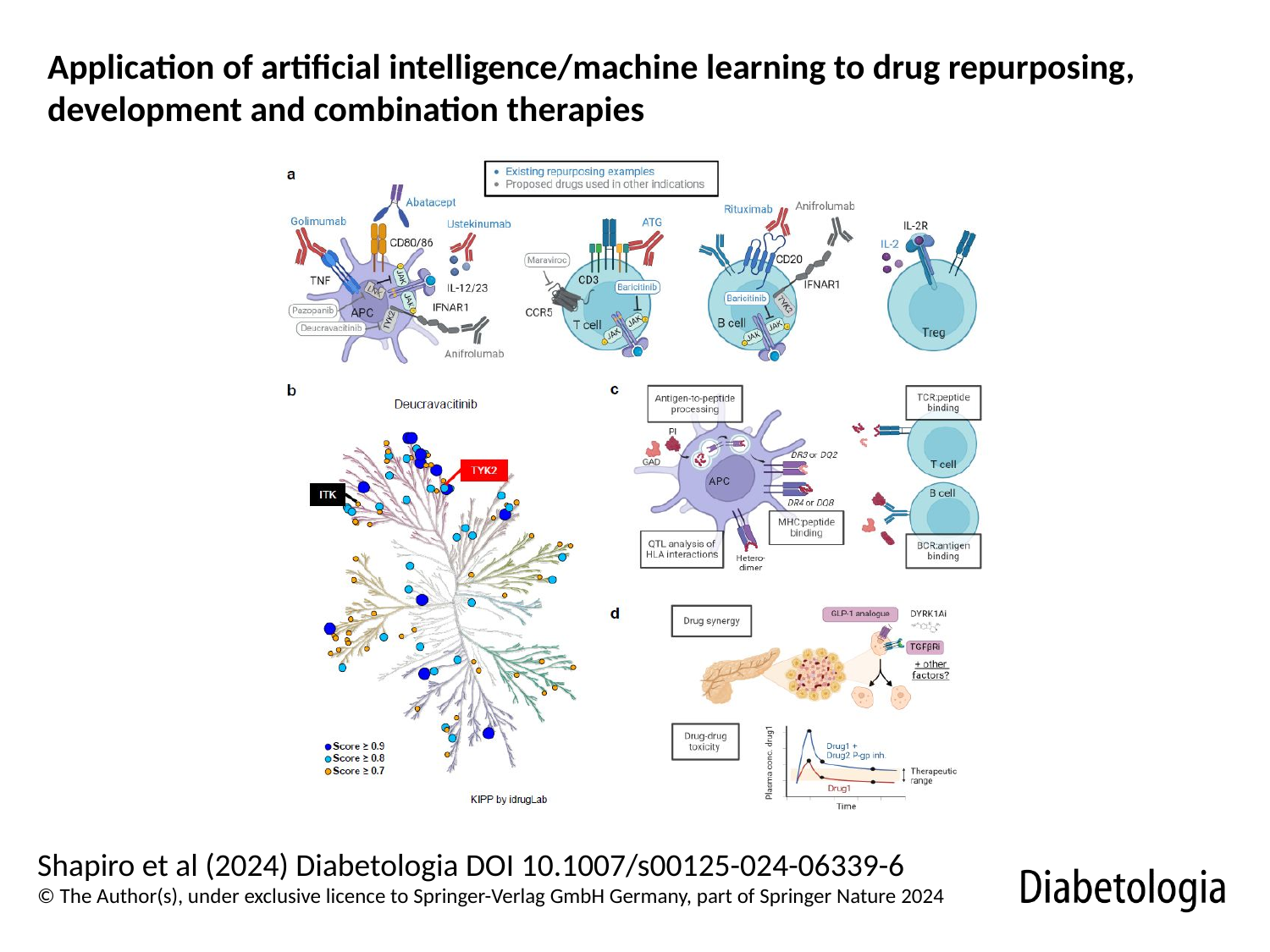

Application of artificial intelligence/machine learning to drug repurposing, development and combination therapies
Shapiro et al (2024) Diabetologia DOI 10.1007/s00125-024-06339-6
© The Author(s), under exclusive licence to Springer-Verlag GmbH Germany, part of Springer Nature 2024

## Slide 3
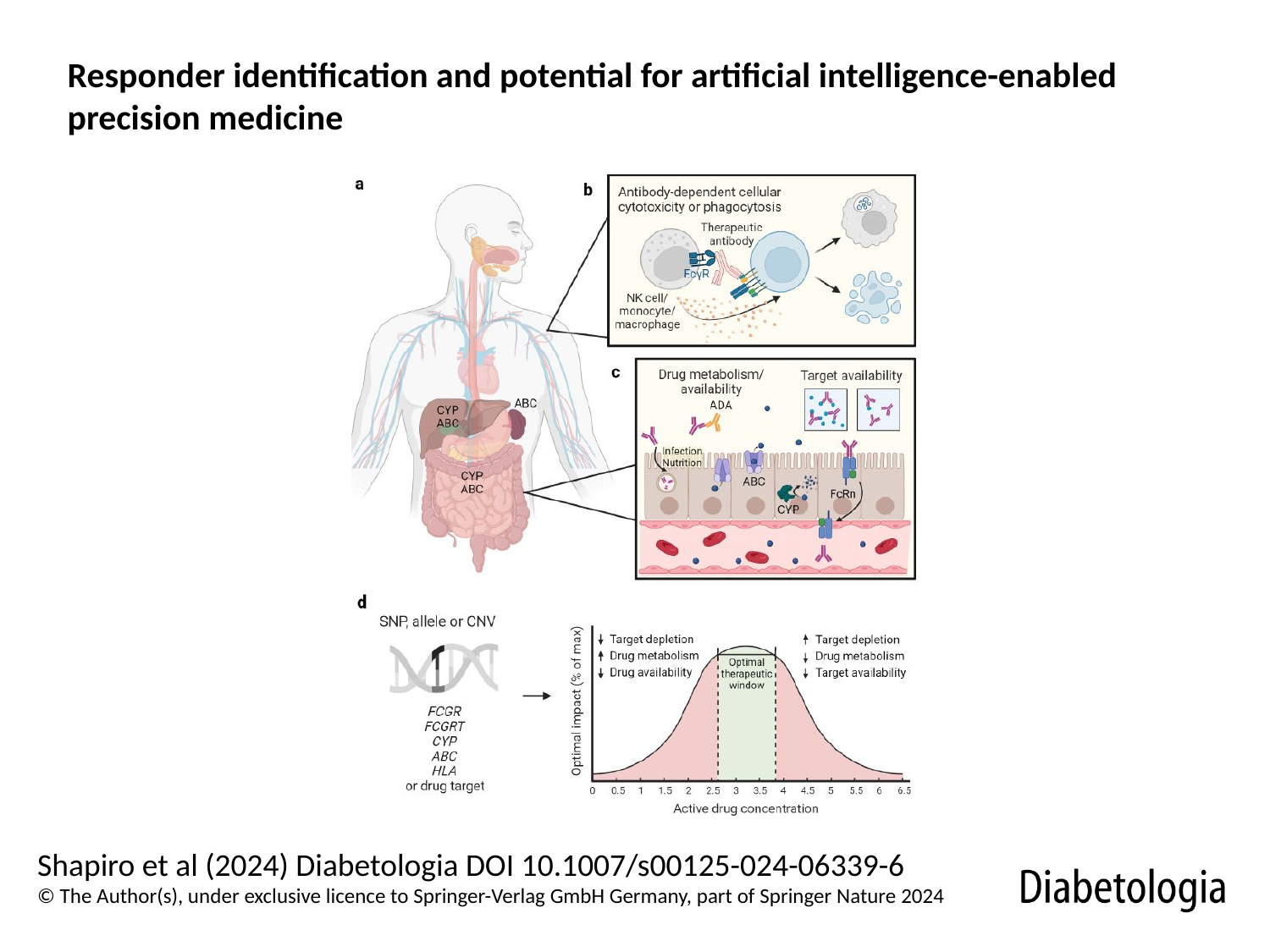

Responder identification and potential for artificial intelligence-enabled precision medicine
Shapiro et al (2024) Diabetologia DOI 10.1007/s00125-024-06339-6
© The Author(s), under exclusive licence to Springer-Verlag GmbH Germany, part of Springer Nature 2024
